# Casbane Diterpene as a Promising Natural Antimicrobial Agent against Biofilm-Associated Infections

**DOI:** 10.3390/molecules16010190

**Published:** 2010-12-30

**Authors:** Victor Alves Carneiro, Hélcio Silva dos Santos, Francisco Vassiliepe Sousa Arruda, Paulo Nogueira Bandeira, Maria Rose Jane Ribeiro Albuquerque, Maria Olívia Pereira, Mariana Henriques, Benildo Sousa Cavada, Edson Holanda Teixeira

**Affiliations:** 1 Department of Biochemistry and Molecular Biology, Faculty of Medicine of Sobral, Federal University of Ceará, Fortaleza, CE, Brazil; E-Mails: bscavada@gmail.com (B.S.C.); edsonlec@gmail.com (E.H.T.); 2 Centre of the Exact Sciences and Technology, Acaraú Valley State University, 62040-370, Sobral, CE, Brazil; E-Mails: helciodossantos@gmail.com (H.S.S.); bandeirapn@yahoo.com.br (P.N.B.); rjane_7@hotmail.com (M.R.J.R.A.); 3 Northeast Biotechnology Network (RENORBIO), State University of Ceará, 60740-000, Fortaleza, CE, Brazil; E-Mail: vassiliepe@gmail.com (F.V.S.A.); 4 Centre for Biological Engineering, IBB-Institute for Biotechnology and Bioengineering, University of Minho, Campus de Gualtar, 4710-057 Braga, Portugal; E-Mails: mopereira@deb.uminho.pt (M.O.P.); mcrh@deb.uminho.pt (M.H.)

**Keywords:** casbane diterpene, biofilm-associated infections control, natural antimicrobials, bacteria and yeast

## Abstract

*Croton nepetaefolius* is a native plant from northeastern Brazil that belongs to the Euphorbiaceae family. The biological action of this plant has been extensively explored, being the secondary metabolites responsible for its properties alkaloids, diterpenes, and triterpenes. This study aimed to evaluate the ability of casbane diterpene (CD), isolated from the ethanolic extract of *C. nepetaefolius*, to inhibit microbial growth and biofilm formation of several clinical relevant species (bacteria and yeasts). It was found that CD possessed biocidal and biostatic activity against the majority of the species screened, with minimal active concentrations ranging between 125 and 500 µg/mL. In addition, it was observed that biofilm formation was inhibited even when the planktonic growth was not significantly affected. In conclusion, CD showed potential to be a natural tool for the treatment of diseases caused by different infectious microorganisms.

## 1. Introduction

In Nature, microorganisms often attach to surfaces and embed themselves in a matrix composed of extracellular polymeric substances, that they properly produce, forming a sessile population called biofilms [1,2]. Moreover, it is known that surface-associated microorganisms exhibited a distinct phenotype with respect to gene transcription, growth rate and enhanced resistance to antimicrobials [3,4].

Biofilms are sources of diverse problems in various areas. In dairy industry, biofilms are often sources of biological contaminants and they also contribute to increased equipment corrosion rates [5]. In the public health sector, the colonization of medical surfaces, such as catheters and other indwelling devices, by biofilms, plays a decisive role in the problem of healthcare-associated infections [6]. Thus, over the years, many efforts have been put on the control of microbial adhesion and biofilm formation [7,8,9,10,11].

Currently, natural products are recognized as important antimicrobial agents with structural and mode of action diversity. Therefore, natural plant compounds have been used by many research groups with the intent of discovering new antimicrobial and anti-biofilm drugs or alternatives to antibiotic therapy [12,13,14]. Several thousand antimicrobial products have been discovered so far, showing high potential for therapeutical use [15].

The genus *Croton* of the plant family Euphorbiaceae is widespread in northeastern Brazil. The use of this genus in opular medicine includes treatments for cancer, constipation, diabetes, digestive problems, dysentery, external wounds, fever, hypercholesterolemia, hypertension, inflammation, intestinal worms, malaria, pain, ulcers, and weight-loss [16]. Previous phytochemical investigations have shown that plants of this genus produce alkaloids [17,18], flavonoids [19,20,21], triterpenoids and steroids [22,23], and a large number of diterpenoids [24,25,26,27,28]. *Croton nepetaefolius* is an aromatic plant native of the Northeast of Brazil which is extensively used in folk medicine as a sedative and antispasmodic agent [29]. Terpenoids are a class of secondary metabolites made of isoprene units. These molecules are reported as possessing antimicrobial properties [30,31], highlighting the antimicrobial potential of this important class of compounds. 

In the present study, the biostatic and biocidal effects of casbane diterpene, a diterpenoid isolated from *Croton nepetaefolius*, was assessed against a wide range of microorganisms, both yeast and bacteria. Moreover, the effects of this compound on biofilm formation was also evaluated. 

## 2. Results and Discussion

**Figure 1:**
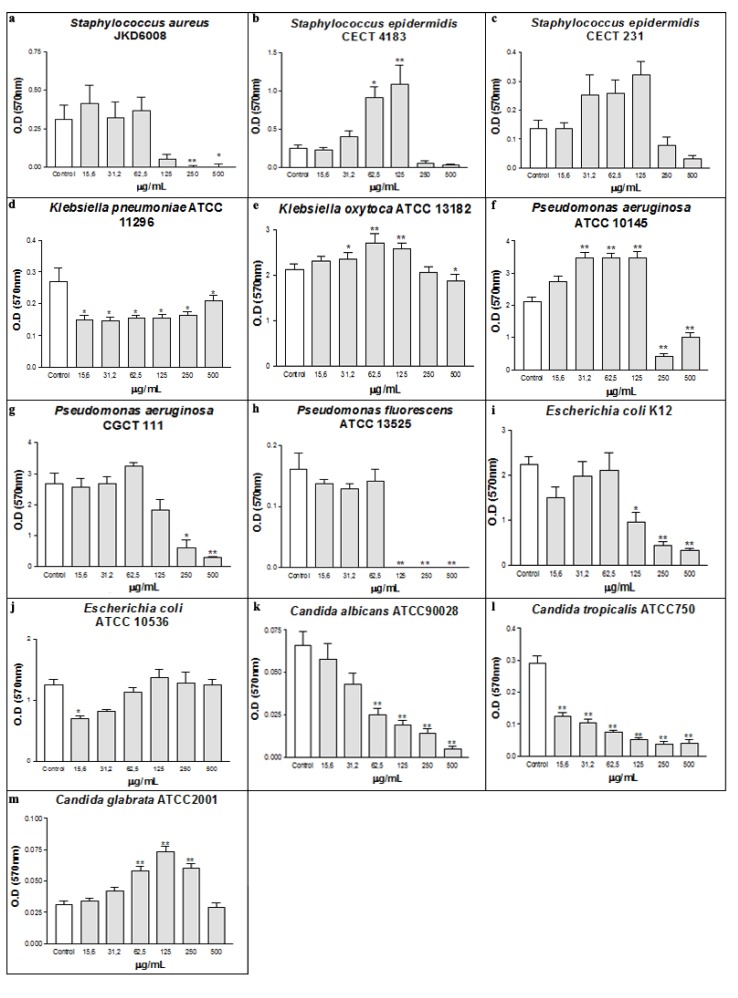
Antimicrobial activity of CD on the planktonic growth of bacterial (a-j) and yeasts (k-m).* p < 0.01 and ** p < 0.001 related to control.

**Figure 2:**
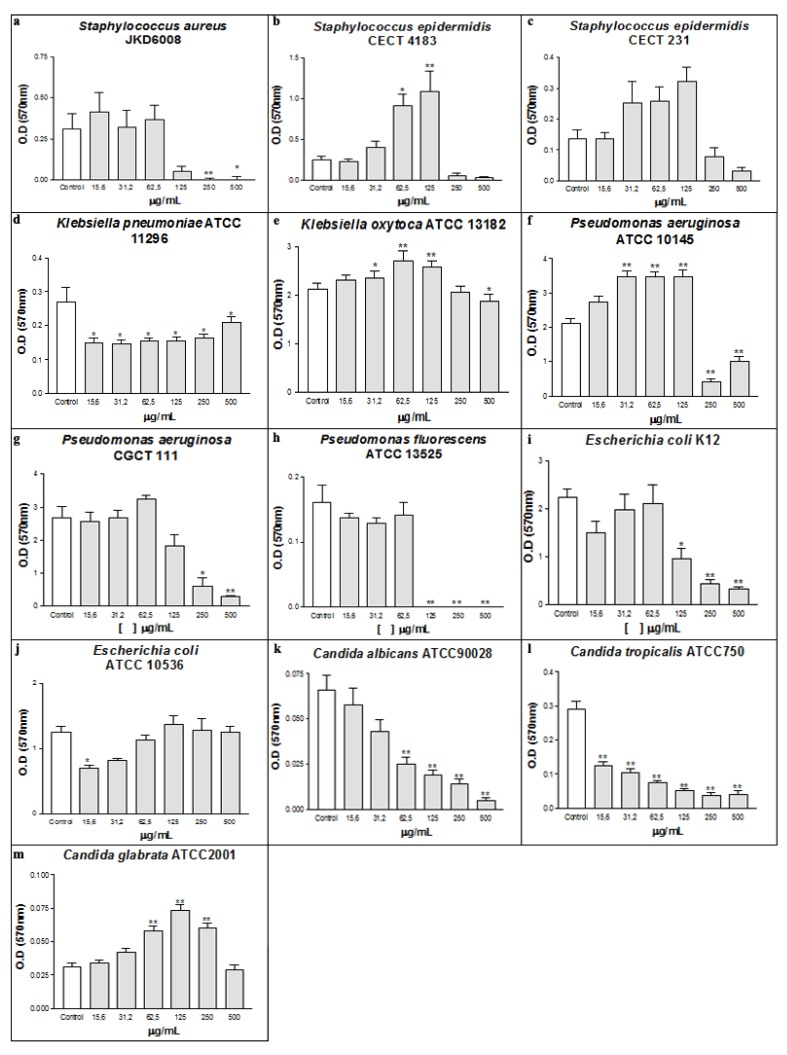
Antibiofilm effect of CD on bacterial (a-j) and yeasts (k-m). *p < 0.01 and ** p < 0.001 related to control.

Bacteria and fungi are widely distributed in Nature, being some of them pathogenic and directly involved in several infectious diseases, such as cystic fibrosis, endocarditis, and periodontitis [32]. The casbane diterpene (CD) fraction extracted from *Croton nepataefolius* showed antimicrobial activity against some microorganisms tested (Figure 1 and Figure 2). The presence of CD during bacteria growth clearly interfered with the Gram-positive bacterial planktonic and sessile development, inhibiting or reducing its growth. Concerning *Staphylococcus aureus*, 125 µg/mL of CD decreased its planktonic growth around 74% according to control absorbance, with MIC 250 µg/mL and MMC 500 µg/mL (Figure 1a). CD also interfered with the establishment of *S. aureus* biofilms, inhibiting their development at concentrations above 125 µg/mL (Figure 2a). 

Regarding *Staphylococcus epidermidis* strains, CD also showed ability to reduce their planktonic growth at concentrations above 62.4 µg/mL, with MIC of 500 µg/mL without bactericidal activity (Figure 1b and Figure 1c). Biofilm formation by *S. epidermidis* CECT 4183 is clearly disturbed by CD at doses above 250 µg/mL (Figure 2b). No significant effect was found on *S. epidermidis* CECT 231 (Figure 2c). So, based on the results it can be concluded that CD exhibited excellent antibacterial profile on the Gram-positive bacteria tested.

As already reported [33,34], the cell membrane has a great importance in many cellular processes, including permeability, cell growth and division. Considering the chemical characteristics of the molecule tested, hidrophobicity and polarity, a non-specific interaction with membrane phospholipids, destabilizing the non-covalent interactions between the fatty acids of the lipid bilayer, and thus interfering with the cellular development of the Gram-positive bacteria can be suggested. Molecules with lipophilic characteristics such as anthraquinones are known antimicrobial substances, which exhibit such an effect of interaction with cell membrane phospholipids [35,36]. The effect on biofilm formation by the Gram-positive staphylococcal strains seems to be directly related to the growth inhibition, showing non-specific action related to the antibiofilm activity.

The effect of CD was different on Gram-negative strains, being able to interfere only in the development of the biofilm, without affecting the planktonic growth, with the exception of *P. fluorescens* ATCC 13525 (Figure 1d). The presence of the outer membrane in Gram-negative bacteria constitutes a barrier for permeability of hydrophobic molecules [37]. Thus, the interaction of CD with the cellular membrane was limited, and the antibacterial effect was inhibited. These microorganisms, when associated in biofilms, are structurally and physiologically different from planktonic bacteria, for example, in their resistance to antibiotics [38].

In recent years, researchers have explored the activity of natural products possessing the ability to interfere with the development of biofilms [39,40]. At the lowest concentration (15.6 µg/mL) biofilm formation by *Klebsiella pneumoniae* ATCC 11296 was decreased by about 45% (Figure 2d). Concerning *Pseudomonas aeruginosa* ATCC 10145, CD at doses ranging between 31.2 and 125 µg/mL induced biofilm mass production, while concentrations above 250 µg/mL strongly inhibited biofilm development by around 80% (Figure 2 f). A similar trend occurred for *Pseudomonas aeruginosa* CGCT 111 and *Escherichia coli* K12 strains, since the highest concentration of CD led to an inhibition of 86% (Figure 2 g and Figure 2i). The increase of biomass observed in the *P. aeruginosa* ATCC10145 can be explained by stress induced by the presence of the tested substance that maybe leads to an extra production of exopolysaccharides (EPS) by the bacterial cell. A similar effect was observed at sub-inhibitory concentrations of cefotaxime, which significantly induced the production of biofilm mass as well as EPS of three *Salmonella enterica* isolates [41]. Against *Pseudomonas fluorescens*, CD showed MIC and MMC of 125 µg/mL and 250 µg/mL, respectively (Figure 1d). Moreover, there was no biofilm formation by this Gram-negative strain at concentrations above 125 µg/mL (Figure 2h). The lipopolysaccharide (LPS) present in the cell surface, placed on outer leaflet of the outer membrane of all Gram-negative bacteria, forms the first point of contact between the bacterial cell and any surface that it colonizes or binding to therapeutic agents [42]. Studies indicate that biofilm formation of *P. aeruginosa* is directly related to the type of LPS produced by the cell [43]. Thus, the effect of CD on inhibition of biofilm formation may be related to an interaction between CD and LPS, which might affect the adherence properties influencing thus biofilm formation by these strains.

As occurred in Gram-positive bacteria, CD was effective on planktonic growth of the yeasts tested. On *C. albicans* and *C. tropicalis* strains CD, at concentrations of 500 µg/mL, reduced viability of planktonic growth in 59% and 29%, respectively (Figure 1k and Figure 1l). However, on *C. glabrata*, the CD was effective in a lower concentration (15.6 µg/mL) and was able to decrease by 72% the yeast viability when its concentration was 500 µg/mL (Figure 1m). In the biofilm formation experiments using yeasts, CD proved to be effective on *C. albicans* and *C. tropicalis* strains, showing a dose-response relationship (Figure 2k and Figure 2l). Regarding *C. tropicalis*, CD at a concentration of 15.6 µg/mL decreased more than 50% the yeast ability to form biofilm (Figure 2l). When used against *C. albicans*, 62.5 µg/mL of the CD was necessary to achieve the same reduction (Figure 2k). On the other hand, CD increased the biomass production of *C. glabrata* (Figure 2m). The differences in CD activity can be explained by differences in phosphoglycerides components and fatty acyl chains of *Candida* species [44].

## 3. Experimental

### 3.1. Plant material

Stalks from *C. nepetaefolius* were collected in Caucaia – Ceará (Brazil) in May 2004. The material was identified by Dr. Edson Paula Nunes at the Herbário Prisco Bezerra (EAC), Departamento de Biologia, Universidade Federal do Ceará, Fortaleza, CE, Brazil, where the voucher specimens (No. 33.582) were deposited. 

### 3.2. Extraction and isolation of casbane diterpene

**Figure 3:**
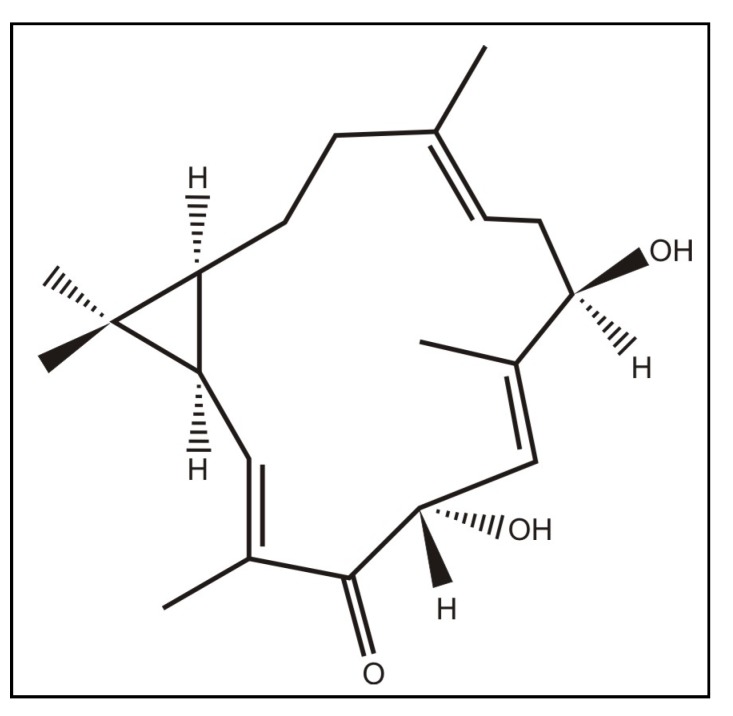
Structure of casbane diterpene extracted from the stalks of *Croton nepetaefolius.*

The bark (5.0 kg) of *C. nepetaefolius* was powdered and extracted with ethanol (EtOH), (10 L × 3, for three days) at room temperature. The solvent was removed under reduced pressure to give an EtOH extract (58.2 g) that was fractionated coarsely on a silica gel column by elution with hexane (fractions 1-15), hexane/ethyl acetate (EtOAc) (1:1 fractions 16-25), EtOAc (fractions 26-40), and EtOH (fractions 41-48), affording a total of 48 fractions of 100 mL each. The hexane fractions (22.5 g) were pooled and fractionated on a silica gel column using hexane (fractions 1^'^-10^'^), hexane/EtOAc (1:1 F^'^ 11-16), EtOAc (F^'^ 17-21) and EtOH (F^'^ 22-25), providing 25 fractions of 100 mL each. Fractions 11^'^-16^'^ (14.0), obtained with hexane/EtOAc (1:1), was fractionated coarsely on a silica gel column by elution with hexane (F^''^ 1), hexane/EtOAc (9:1 F^''^ 2-5; 8:2 F^''^ 6-15; 7:3 F^''^ 16-32), EtOAc (F^''^ 33), providing 33 fractions of 100 mL each. Fractions 10^''^-13^''^, obtained with hexane/EtOAc (8:2), yielded diterpene named1, 4-dihydroxy-2E,6E,12E-trien-5-one-casbane (3.0 g, 0.06%; Figure 3).

### 3.3. Preparation of the CD stock solution

The CD was solubilized in dimethyl sulfoxide (DMSO), and then diluted in culture medium (1 mg/mL), with a maximum percentage of 4% of DMSO. Controls were performed to confirm that this dose of DMSO did not interfere with microbial growth.

### 3.4. Microorganisms

In the present study, the microorganisms used in the experiments were: Gram negative bacteria (*Pseudomonas fluorescens* ATCC13525, *Pseudomonas aeruginosa* ATCC10145, *P. aeruginosa* CGCT111, *Klebsiella oxytoca* ATCC13182, *Klebsiella pneumoniae* ATCC11296, *Escherichia coli* K12 MG 1655, and *Escherichia coli* CECT434), Gram-positive bacteria (*Staphylococcus epidermidis* CECT231, *S. epidermidis* CECT4183, and *S. aureus* ATCC), and yeasts (*Candida albicans* ATCC90028*, C. tropicalis* ATCC750 and *C. glabratta* ATCC2001).

### 3.5. Culture conditions

For each microorganism, a culture stock was prepared on Tryptic Soy broth (TSB) plus 20% glycerol and preserved at -80 °C. Then, the microorganisms were transferred into Petri dishes containing TSA and incubated at 37 °C, for 24 h. After growth on the solid medium, an isolated colony was removed and inoculated into 10 mL of TSB and incubated for 18 h at 37 °C under constant agitation of 120 rpm. Prior to use, the cell concentration of each inoculum was adjusted to 1 × 10^6^ cells/mL through the use of spectrophotometer and calibration curves, previously determined for each bacterium. The yeasts were cultured for 24 h in RPMI 1640 buffered with MOPS at pH 7.0 in constant agitation of 120 rpm. Then, the concentration of each yeast inoculum was adjusted to 1 × 10^6^ cells/mL using a Neubauer chamber.

### 3.6. Antimicrobial assays

The antimicrobial effects of CD were determined by the broth microdilution method in 96-well polystyrene plates, according to the guideline *Methods for Dilution Antimicrobial Susceptibility Tests for Bacteria That Grow Aerobically*; *Approved Standard – Sixth Edition* (NCCLS document M7-A6). CD was diluted in culture medium, RPMI 1640 for yeast or TSB for bacteria, to achieve 15.6 to 500 µg/mL were incubated aerobically with 1 × 10^6^ cells/mL, initial concentration of cells, on a horizontal shaker (120 rpm/min), at 37 °C, during 24 h. The optical density at 640 nm (OD_640_) of each well content was recorded using an automated Elisa Reader (Synergy TM HT Multi-Detection Microtiter Reader), as a measure of microbial growth. 

The minimum inhibitory concentration (MIC) for each microorganism was determined as the lowest concentration of CD at which there is complete inhibition of visible growth of the organism. To determine the minimum microbicidal concentration (MMC), 10 μL of the bacteria/yeast planktonic cultures, where no visible microbial growth was observed, were inoculated in petri dishes with TSA medium and incubated at 37 °C for 24 h. MMC was considered as being the lowest concentration able to completely inhibit microbial growth on the plates.

### 3.7. Antibiofilm activity

The methodology used to grow biofilms was based on the microtiter plate test developed by Stepanovic *et al*. [45], with some modifications. Sterile 96-well polypropylene tissue culture plates (Orange Scientific, Braine-l’Alleud, Belgium) (with flat-bottom) were prepared using a procedure similar to the one used in the antimicrobial activity tests with same initial concentration of cells. All the plates were incubated aerobically on a horizontal shaker (120 rpm/min), at 37 °C during 24 h for biofilm development. After biofilm growth in the presence or absence of same CD concentrations, the content of each well was removed and the biofilms were washed twice with 200 µL/well of sterilized water, to remove cells weakly adhered, being reserved for posterior analysis. 

*Biomass quantification:* The attached biofilm mass was quantified using crystal violet staining [46]. Briefly, the plates containing the biofilms were let to air dry for 30 min, and 200 µL of absolute methilic alcohol were transferred to each well, in order to fix the adhered cells, and allowed to contact during for 15 min. After 15 min, the methanol was removed and 200 µL of crystal violet 1% (Gram colour-staining set for microscopy; Merck) per well were added for 5 min. After the staining step, the washing process, with sterile water, was repeated and the plates were placed at room temperature for 1 hour. To re-solubilize the dye bounded to biofilms, 200 µL of 33% (v/v) glacial acetic acid (Merck) was added to each well and place in agitation for 15 min. The CV solutions obtained were transferred to a new sterile 96-well plate and the optical density of the content of each well was measured using a microtiter plate spectrophotometer (Sunrise - Tecam) at 570 nm.

### 3.8. Statistical analysis

Statistical analyses were performed by GraphPad Prism® version 3.00 from Microsoft Windows®. The method used was one-way ANOVA with Bonferroni *post hoc* test. The data were obtained in triplicates from at least three separate experiments. The graphs were presented as mean ± standard deviation. The data were considered significant when p < 0.01 or p < 0.001.

## 4. Conclusions

Biofilm eradication is a crucial step for the treatment of various diseases. In the present work, a novel and natural agent with a promising antimicrobial activity was described. Casbane diterpene (CD) showed antimicrobial effect on planktonic forms and biofilm from some bacteria and yeasts. The results showed that CD can be considered as a promising molecule with potential for the pharmacological treatment of biofilm-associated infections. Additional toxicological studies need to be performed to validate its applicability.

## Supplementary Materials

Supplementary File 1
