# Supplementary material for: Casbane Diterpene as a Promising Natural Antimicrobial Agent against Biofilm-Associated Infections
Source: Molecules. 2010 Dec 30;16(1):190–201. doi: 10.3390/molecules16010190 (PMC6259237; doi:10.3390/molecules16010190)
Supplement: Supplementary File 1 [file molecules-16-00190-s001.pdf]

Molecules **2011**, *16*

OPEN ACCESS

molecules

ISSN 1420-3049

www.mdpi.com/journal/molecules

Correction

Carneiro *et al*. Casbane Diterpene as a Promising Natural Antimicrobial Agent against Biofilm-Associated Infections. *Molecules*, *16*, 190-201

Victor Alves Carneiro [1,*], Hélcio Silva dos Santos [2], Francisco Vassiliepe Sousa Arruda [3], Paulo Nogueira Bandeira [2], Maria Rose Jane Ribeiro Albuquerque [2], Maria Olívia Pereira [4], Mariana Henriques [4], Benildo Sousa Cavada [1] and Edson Holanda Teixeira [1]

[1] Department of Biochemistry and Molecular Biology, Faculty of Medicine of Sobral, Federal University of Ceará, Fortaleza, CE, Brazil; E-Mails: bscavada@gmail.com (B.S.C.); edsonlec@gmail.com (E.H.T.)

[2] Centre of the Exact Sciences and Technology, Acaraú Valley State University, 62040-370, Sobral, CE, Brazil; E-Mails: helciodossantos@gmail.com (H.S.S.); bandeirapn@yahoo.com.br (P.N.B.); rjane_7@hotmail.com (M.R.J.R.A.)

[3] Northeast Biotechnology Network (RENORBIO), State University of Ceará, 60740-000, Fortaleza, CE, Brazil; E-Mail: vassiliepe@gmail.com (F.V.S.A.)

[4] Centre for Biological Engineering, IBB-Institute for Biotechnology and Bioengineering, University of Minho, Campus de Gualtar, 4710-057 Braga, Portugal; E-Mails: mopereira@deb.uminho.pt (M.O.P.); mcrh@deb.uminho.pt (M.H.)

* Author to whom correspondence should be addressed; E-Mail: victorcarneiro@ufc.br; Tel.: +55-88-3611-8000; Fax: +55-88-3611-2202.

Received: 26 May 2011 / Published: 30 May 2011

The authors wish to make the following correction to this paper [1]:

The Figure 1 was corrected as below:

Figure 1. Antimicrobial activity of CD on the planktonic growth of bacterial (a-j) and yeasts (k-m). *p < 0.01 and **p < 0.001 related to control.

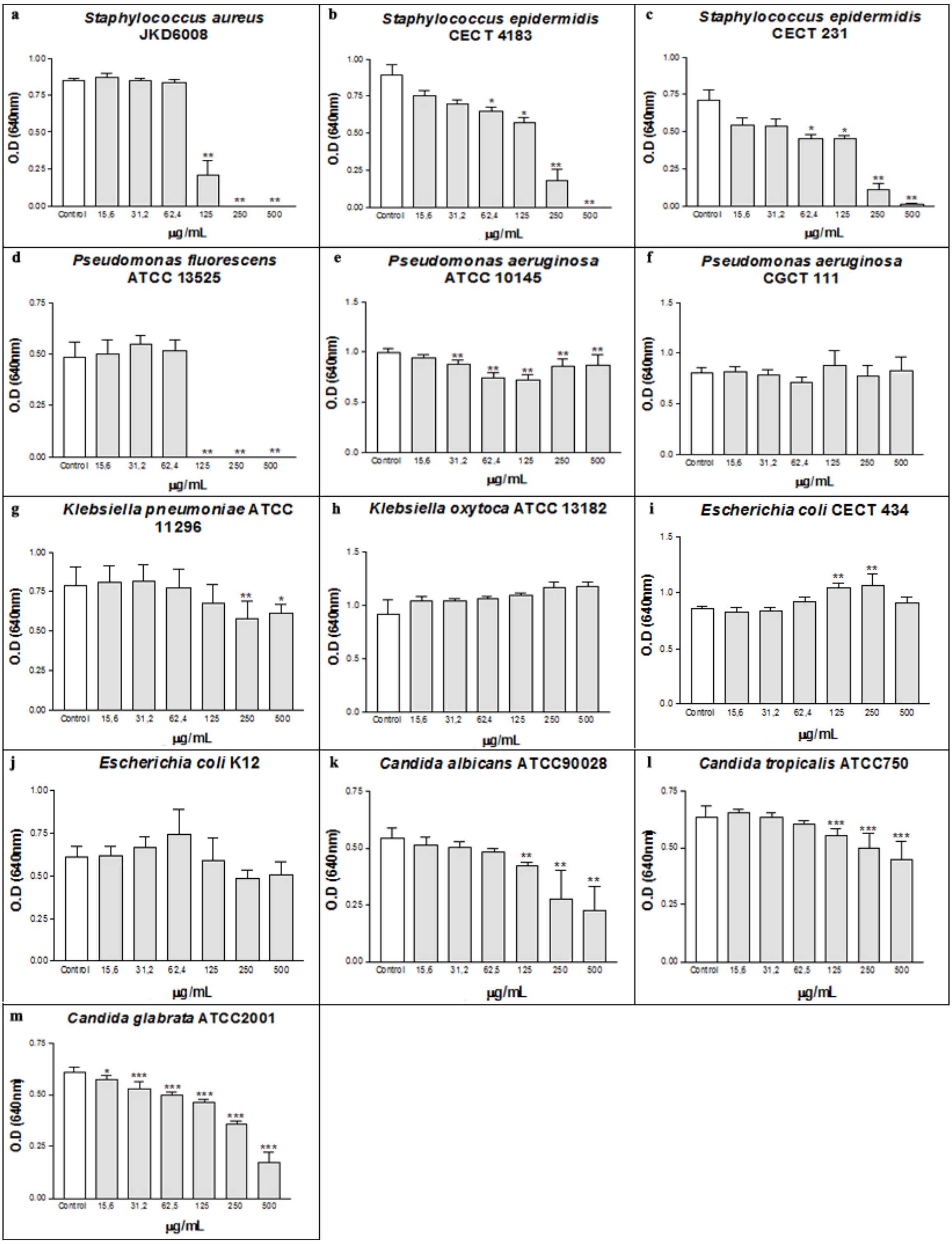

Reference

1. Carneiro, V.A.; Santos, H.S.; Arruda, F.V.S.; Bandeira, P.N.; Albuquerque, M.R.J.R.; Pereira, M.O.; Henriques, M.; Cavada, B.S.; Teixeira, E.H. Casbane Diterpene as a Promising Natural Antimicrobial Agent against Biofilm-Associated Infections. *Molecules* **2011**, *16*, 190-201.

© 2011 by the authors; licensee MDPI, Basel, Switzerland. This article is an open access article distributed under the terms and conditions of the Creative Commons Attribution license (http://creativecommons.org/licenses/by/3.0/).
